# Regulatory role of non-coding RNAs in 5-Fluorouracil resistance in gastrointestinal cancers

**DOI:** 10.20517/cdr.2024.167

**Published:** 2025-01-16

**Authors:** Heng Zhang, Hailin Tang, Wenling Tu, Fu Peng

**Affiliations:** ^1^Department of Pharmacology, West China School of Pharmacy, Sichuan University, Chengdu 610051, Sichuan, China.; ^2^Department of Nuclear Medicine, The Second Affiliated Hospital of Chengdu Medical College, China National Nuclear Corporation 416 Hospital, Chengdu 610051, Sichuan, China.; ^3^State Key Laboratory of Oncology in South China, Guangdong Provincial Clinical Research Center for Cancer, Sun Yat-Sen University Cancer Center, Guangzhou 510700, Guangdong, China.; ^4^Key Laboratory of Drug-Targeting and Drug Delivery System of the Education Ministry, Sichuan Engineering Laboratory for Plant-Sourced Drug and Sichuan Research Center for Drug Precision Industrial Technology, Sichuan University, Chengdu 610041, Sichuan, China.

**Keywords:** Non-coding RNAs, 5-fluorouracil, gastrointestinal cancers, chemoresistance, tumor microenvironment

## Abstract

Gastrointestinal (GI) cancers are becoming a growing cause of morbidity and mortality globally, posing a significant risk to human life and health. The main treatment for this kind of cancer is chemotherapy based on 5-fluorouracil (5-FU). However, the issue of 5-FU resistance is becoming increasingly prominent, which greatly limits its effectiveness in clinical treatment. Recently, numerous studies have disclosed that some non-coding RNAs (ncRNAs), including microRNAs (miRNAs), long non-coding RNAs (lncRNAs), and circular RNAs (circRNAs), exert remarkable physiological functions within cells. In addition, these ncRNAs can also serve as important information communication molecules in the tumor microenvironment and regulate tumor chemotherapy resistance. In particular, they have been shown to play multiple roles in regulating 5-FU resistance in GI cancers. Herein, we summarize the targets, pathways, and mechanisms involved in regulating 5-FU resistance by ncRNAs and briefly discuss the application potential of ncRNAs as biomarkers or therapeutic targets for 5-FU resistance in GI cancers, aiming to offer a reference to tackle issues related to 5-FU resistance.

## INTRODUCTION

Gastrointestinal (GI) cancers constitute a group of prevalent malignant tumors, predominantly encompassing colorectal cancer (CRC), gastric cancer (GC), pancreatic cancer (PC), hepatocellular carcinoma (HCC), cholangiocarcinoma (CCA), and esophageal cancer (EC)^[1]^. GI cancers account for 25% and 30% of all cancers in terms of incidence and mortality, respectively, with the highest incidence rates for CRC and GC and the highest mortality rates for HCC and GC. Globally, East Asia, particularly China, has the highest incidence and mortality rates for GI cancers, which represents a major socio-economic and public health challenge^[2,3]^.

The main treatments for GI cancers include surgery^[4]^, radiotherapy^[5]^, immunotherapy^[6]^, gene therapy^[7,8]^, and therapy targeting cancer stem cells (CSC)^[9]^. However, chemotherapy is still the most effective first-line regimen for treating GI cancers^[10,11]^. 5-fluorouracil (5-FU) represents a heterocyclic aromatic organic compound falling within the category of uracil analogs, possessing a molecular structure analogous to that of pyrimidines present in DNA and RNA^[12]^. 5-FU exerts anticancer effects mainly by disrupting DNA synthesis and repair through repression of thymidylate synthase (TYMS) activity, or by damaging RNA and DNA through incorporation of fluorouridine triphosphate (FUTP) and fluorodeoxyuridine triphosphate (FdUTP), which are produced by the metabolism of 5-FU, into RNA and DNA, respectively^[13,14]^. In addition, factors such as dihydropyrimidine dehydrogenase (DPYD) and thymidine phosphorylase (TP) are also engaged in the regulation of the anticancer activity of 5-FU^[14]^. However, although 5-FU is powerful in treating cancer, its clinical use in the treatment of GI cancers is severely limited by the progression of drug resistance^[13]^. Therefore, understanding the relevant molecular mechanisms of 5-FU resistance in GI cancers is key to solving the resistance problem and improving its efficacy.

Non-coding RNAs (ncRNAs) are a heterogeneous group of transcripts that are generally not engaged in coding for proteins^[15]^. NcRNAs can be broadly categorized into two main types according to their functional characteristics: housekeeping RNAs that are mainly engaged in the maintenance of basic cellular functions, such as tRNAs and rRNAs, and regulatory RNAs that are implicated in the regulation of a variety of biological processes in the cell, such as miRNAs, lncRNAs, circRNAs, siRNAs, and piRNAs^[16]^. NcRNAs play an important regulatory role at various levels of gene expression and thus control the normal physiological function of cells or the occurrence of certain diseases^[17]^. For example, ncRNAs can regulate cell proliferation, differentiation, transcription, post-transcriptional modification, apoptosis, and cell metabolism by interacting with other biomolecules such as DNA, RNA, and proteins^[18,19]^. More importantly, ncRNAs have been proved to be crucial in tumor development and participate in cancer cell proliferation, migration, and invasion. NcRNAs also exhibit a close correlation with the emergence of chemotherapy resistance in tumors, particularly the development of 5-FU chemotherapy resistance^[20,21]^. It is worth noting that ncRNAs also serve as important information communication molecules in the tumor microenvironment (TME); thus, they could be used by tumor cells to specifically affect both cellular and non-cellular components of TME and create a microenvironment suitable for tumor growth^[22,23]^. Additionally, ncRNAs can also become one of the powerful tools for tumor cells to shape the drug resistance microenvironment by participating in the regulation of specific pathways, thus becoming the important factor that affects the tumor cells to respond to chemotherapy drugs^[24,25]^.

The pivotal regulatory roles of ncRNAs, along with the diversity of their interactions, contribute to an increasingly extensive and complex regulatory network. This complexity indicates that ncRNAs can serve as critical regulators in modulating essential intracellular programs^[26]^. Many studies have demonstrated that certain ncRNAs can participate in the modulation of chemotherapeutic drug resistance by influencing the epithelial-mesenchymal transition (EMT) process, extracellular hypoxia microenvironment, cell apoptosis, and other pathways, which has been confirmed in GI cancers^[27-31]^. However, there is still a lack of a systematic summary of the ncRNAs related to 5-FU resistance and its regulatory mechanism. Therefore, this paper mainly summarizes miRNAs, lncRNAs, and circRNAs related to 5-FU resistance in GI cancers, reviews their regulatory mechanisms, and additionally discusses some applied research on ncRNAs in the article, hoping to provide some references to solve this problem.

## NCRNAS INFLUENCE 5-FU RESISTANCE IN GI CANCERS

### 5-FU resistance in GI cancers

As an antimetabolite with numerous mechanisms and excellent efficacy, 5-FU occupies an important position in cancer treatment, particularly in the context of treating GI cancers. For example, the FOLFOX regimen or FOLFIRI regimen is the first line of therapy in the treatment of CRC^[32]^. The combination of 5-FU with irinotecan, docetaxel, or oxaliplatin represents an efficacious alternative for the treatment of GC as well^[33]^. There is no doubt that 5-FU, as a representative chemotherapeutic agent, has an important position in cancer treatment, especially in the treatment of GI cancers, and is also one of the basic drugs in many combination therapy programs^[34]^.

However, the development of primary and secondary resistance has become a common phenomenon in 5-FU chemotherapy since its widespread application, as well as in GI cancers. Therefore, it is urgent to discover the molecules and mechanisms involved in the generation of drug resistance^[35]^. Many factors can contribute to the progression of 5-FU resistance, such as TYMS overexpression, resistance to apoptosis, enhanced autophagy, EMT pathway, increased efflux of drugs mediated by the family of transporter proteins, increased activity of the dihydropyrimidine dehydrogenase (DPD), the generation of DNA mismatch repair defects, and the hypoxic microenvironment in tumor tissues^[13,36]^. In conclusion, the development and regulation of 5-FU resistance is a multifactorial event.

Currently, the new generation of fluoropyrimidine (FP) polymers with lower anabolic dependence and higher TS-restrained activity excel in addressing the problem of acquired 5-FU resistance, and its second-generation FP drug, CF10, overcomes the emergence of resistance by hindering TS and DNA topoisomerase 1 (Top 1)^[37]^. In addition, the development of new strategies such as the use of MDR reversal agents, DNA repair suppression for CSC, DPD inhibition, and downregulation of the Bcl-XL protein are all effective reversal strategies for 5-FU chemoresistance^[13,38]^.

### NcRNAs in GI cancers

In GI cancer cells, different types of ncRNAs have different functions. MiRNAs are capable of interacting with the 3’-UTR portion of target mRNAs, thereby eliciting their post-transcriptional repression^[39]^. LncRNAs can engage in transcriptional regulatory processes and act as competitive endogenous RNAs (ceRNAs) to sponge miRNAs. This interaction modulates mRNA-miRNA binding, thereby regulating the expression of target genes^[18]^. In addition, lncRNAs can also directly bind to protein or nucleic acid molecules to play a regulatory role in tumor cells^[40]^. CircRNAs can control certain transcription processes in the nucleus, or act as miRNA sponges and compete with other mRNAs to bind miRNAs^[41]^. SiRNAs can bind to target mRNA and promote its degradation in a completely complementary pairing manner, resulting in silencing of target genes. The targeted delivery technology of siRNA drugs has been widely used in the treatment of GI cancers^[42]^. PiRNAs modulate the gene silencing pathway by binding with Piwi subfamily proteins to constitute the piRNA complex (piRC). This complex plays a crucial role in either promoting or repressing GI cancers^[43]^.

Many ncRNAs are involved in the functional regulation of cancer cells, including GI cancer cells, forming a network that drives specific cellular processes by influencing multiple molecular targets or pathways^[26]^. For example, Ashrafizadeh *et al*. showed that ncRNAs could influence cancer progression and treatment response by adjusting the signal transducer and activator of the transcription 3 (STAT3) pathway in GI cancers^[44]^. In addition, Xu *et al*. revealed that ncRNAs could affect the outcome of radiation therapy for tumors by targeting specific targets or pathways and thus acting as radiosensitivity enhancers or radiotherapy resistance inducers in GI cancers^[45]^. In summary, ncRNAs can exert specific regulatory functions on GI tumors by directly or indirectly interfering with gene expression, and exert a significant influence on the emergence and progression of GI cancers^[46-50]^.

### ncRNAs regulate 5-FU drug resistance in GI cancers

Overcoming 5-FU resistance has become a central focus of concern, and the function of ncRNAs in regulating tumor chemoresistance is also being investigated. It has been demonstrated that ncRNAs could participate in the modulation of 5-FU chemoresistance by modulating cell apoptosis, TS level, cell autophagy, cell cycle, adenosine triphosphate-binding cassette (ABC) transporter proteins, and CSCs^[51]^. In addition, as one of the regulatory factors in the TME, ncRNAs can affect the interaction between cancer cells and TME by orchestrating gene expression in tumor cells and shaping the drug-resistant TME^[52]^, which provides a new perspective for solving the problem of 5-FU resistance. The important regulatory role of ncRNAs suggests that they may have great potential in overcoming 5-FU chemotherapy resistance. Many studies have demonstrated that ncRNAs, including miRNAs, lncRNAs, and circRNAs, all take part in the regulation of 5-FU resistance in GI cancers, which include CRC^[30]^, GC^[53]^, HCC^[27]^, PC^[54]^, EC^[31]^, and CCA^[55]^. Herein, this assay focuses on ncRNAs involved in 5-FU chemotherapy in GI cancers and briefly summarizes their regulatory targets and mechanisms.

## NCRNAS PROMOTE 5-FU RESISTANCE IN GI CANCERS

### MiRNAs that promote 5-FU resistance in GI cancer

In most cases of GI cancers, miRNAs have been found to be overexpressed or upregulated, thus enhancing the chemoresistance of GI cancer cells to 5-FU [Table 1]. These miRNAs can bind to specific mRNAs and downregulate their expression, ultimately promoting 5-FU resistance through multiple pathways in different cancer types^[56-77]^.

**Table 1:** MiRNAs that promote 5-FU resistance in GI cancers

**miRNAs**	**Cancer type**	**Expression**	**Target or pathway**	**Ref.**
miR-10b	CRC	up	BIM	[56]
miR-23a	CRC	up	APAF1	[57]
miR-23a	CRC	up	ABCF1	[58]
miR-675-5p	CRC	up	HIF-1α	[60]
miR-106a	CRC	up	DUSP2	[61]
miR-196b-5p	CRC	up	SOCS1, SOCS3	[62]
miR-195	CRC	up	CHK1, WEE1	[63]
miR-200a-3p	HCC	up	DUSP6	[64]
miR-122	HCC	up	PCDH20	[65]
miR-141	HCC	up	KEAP1	[66]
miR-181c	PC	up	MST1, LATS2, SAV1, MOB1A	[68]
miR-320a	PC	up	PDCD4	[69]
miR-21	PC	up	PDCD4, PTEN	[70]
miR-183	PC	up	PTEN	[71]
miR-499a-5p	PC	up	PTEN	[72]
miR-221-3p	PC	up	RB1	[73]
miR-130b	GC	up	CMPK1	[74]
miR-4516	GC	up	ING4	[75]
miR-1229-3p	GC	up	TYMS, DPYD,	[76]
miR-221	EC	up	DKK2	[77]

5-FU: 5-fluorouracil; GI: gastrointestinal; miRNAs: microRNAs; CRC: colorectal cancer; PC: pancreatic cancer; HCC: hepatocellular carcinoma; GC: gastric cancer; EC: esophageal cancer; BIM: Bcl-2 interacting mediator of cell death; APAF-1: apoptotic protease activating factor 1; ABCF1: ATP binding cassette subfamily F member 1; HIF-1α: hypoxia-inducible factor-1a; DUSP2: dual specificity phosphatase 2; SOCS1: suppressor of cytokine signaling 1; SOCS3: suppressor of cytokine signaling 3; CHK1: checkpoint kinase 1; WEE1: WEE1 G2 checkpoint kinase; DUSP6: dual-specificity phosphatase 6; PCDH20: protocadherin 20; KEAP1: Kelch-like ECH-associated protein 1; MST1: macrophage stimulating 1; LATS2: large tumor suppressor kinase 2; SAV1: salvador family WW domain containing protein 1; MOB1A: MOB kinase activator 1A; PDCD4: programmed cell death factor 4; PTEN: phosphatase and tensin homolog; RB1: RB corepressor 1; CMPK1: cytidine/uridine monophosphate kinase 1; ING4: Inhibitor of growth family member 4; DKK2: dickkopf WNT signaling pathway inhibitor 2.

#### CRC

The Bcl-2-interacting mediator of cell death (BIM) potently induces apoptosis by repressing the anti-apoptotic members of the B-cell lymphoma-2 (Bcl-2) family. However, miR-10b can directly target the mRNA of BIM to reduce its expression, thereby inhibiting apoptosis of CRC cells and promoting the emergence of 5-FU resistance^[56]^. The apoptotic factor caspase-9 is positively regulated by apoptotic protease-activating factor 1 (APAF1) in CRC cells. Shang *et al*. discovered that miR-23a impeded 5-FU-triggered apoptosis within CRC cells by negatively regulating the APAF1/caspase-9 pathway^[57]^. Furthermore, Li *et al*. demonstrated that the elevated expression of miR-23a in microsatellite instability CRC cells likewise augmented cellular chemoresistance to 5-FU by directly targeting ATP binding cassette subfamily F member 1 (ABCF1)^[58]^.

A hypoxic TME is commonly found in solid tumors and is closely associated with chemoresistance. Tumor cells can acclimate to the hypoxic TME predominantly through the upregulation of hypoxia-inducible factor-1α (HIF-1α)^[59]^. It was confirmed that prolonged hypoxia could increase the level of miR-675-5p, which maintains hypoxic responses by regulating HIF-1α mRNA stability, supporting hypoxia-induced 5-FU resistance in CRC cells^[60]^. Qin *et al*. discovered that miR-106a could downregulate the expression of dual specificity phosphatase 2 (DUSP2), leading to increased expression of genes that maintain cancer cell stemness^[61]^. It was manifested that miR-196b-5p could instigate the STAT3 signaling pathway by targeting the suppressor of cytokine signaling 1 (SOCS1) and suppressor of cytokine signaling 3 (SOCS3), which are negative regulators of the STAT3 pathway in CRC cells, thereby enhancing chemoresistance to 5-FU^[62]^. Kim *et al*. revealed that miR-195 could increase the 5-FU resistance of CRC cells by directly targeting checkpoint kinase 1 (CHK1) and WEE1 G2 checkpoint kinase^[63]^.

#### HCC

In HCC cells, a high level of miR-200a-3p would lead to increased 5-FU resistance. Mechanistically, miR-200a-3p can restrain the 5-FU-induced apoptotic effects by downregulating dual-specificity phosphatase 6 (DUSP6), a member of the mitogen-activated protein kinase (MAPK) family^[64]^. MiR-122 can increase the phosphorylation of protein kinase B (Akt) by reducing the expression of protocadherin 20 (PCDH20), which in turn leads to an increase in mTOR activity and ultimately causes the resistance of HCC cells to 5-FU^[65]^. Shi *et al*. reported that the binding interaction between miR-141 and kelch-like ECH-associated protein 1 (KEAP1) instigated the nuclear translocation of nuclear factor erythroid-2-related factor-2 (Nrf2) and the subsequent activation of the Nrf2-dependent antioxidant signaling pathway. This cascade of events ultimately enhanced the resistance of HCC to 5-FU^[66]^.

#### PC

The Hippo signaling pathway is closely linked to the chemotherapy tolerance of tumors, and suppression of the Hippo signaling pathway would exacerbate this problem^[67]^. Overexpression of miR-181c can inactivate the Hippo signaling pathway via directly targeting macrophage stimulating 1 (MST1), large tumor suppressor kinase 2 (LATS2), salvador family WW domain containing protein 1 (SAV1), and MOB kinase activator 1A (MOB1A), resulting in overactivation of yes-associated protein (YAP)/transcriptional coactivator with PDZ-binding motif (TAZ) and promoting 5-FU chemoresistance in PC cells^[68]^. In another investigation, Wang *et al*. ascertained that miR-320a exhibited a marked upregulation in 5-FU-resistant PC cells. It was further demonstrated that miR-320a could bind to the mRNA of programmed cell death factor 4 (PDCD4) and negatively regulate its expression, thereby promoting 5-FU resistance in PC cells^[69]^. Similarly, Wei *et al*. found that miR-21 was also able to induce 5-FU resistance in PC cells by downregulating the expression of PDCD4 and phosphatase and tensin homolog (PTEN)^[70]^. In addition, miR-183 and miR-499a-5p were also able to suppress PTEN expression, thereby activating the PI3K/Akt signaling pathway and promoting the tolerance of PC to 5-FU chemotherapy^[71,72]^. The upregulated expression of miR-221-3p could also augment the resistance of PC cells toward 5-FU. This is because miR-221-3p could downregulate the expression of the transcriptional retinoblastoma corepressor 1 (RB1), thereby promoting the process of EMT^[73]^.

#### Other cancer types

In GC cells, cytidine/uridine monophosphate kinase 1 (CMPK1) is an important regulatory enzyme for the conversion of 5-FU into cytotoxic metabolites. Chu *et al*. discovered that miR-130b could negatively regulate the expression of CMPK1 in GC, resulting in resistance to apoptosis after treatment with 5-FU^[74]^. Overexpression of miR-4516 could downregulate the expression of its target gene inhibitor of growth family member 4 (ING4) and thereby enhance chemoresistance of GC cells through the elevation of Bcl-2 expression or the reduction of Bcl-2 associated X (BAX) and caspase-3 expression^[75]^. Nishibeppu *et al*. identified that the overexpressed miR-1229-3p caused the upregulation of TYMS and DPYD, resulting in resistance of GC cells to 5-FU^[76]^.

As for EC cells, Wang *et al*. confirmed that increased expression of dickkopf WNT signaling pathway inhibitor 2 (DKK2) was able to inactivate the Wnt/β-catenin signaling pathway and dysregulation of chemoresistance target genes in EC cells. However, overexpression of miR-221 could elevate 5-FU resistance by restraining DKK2 expression^[77]^.

### LncRNAs that promote 5-FU resistance in GI cancers

It has been confirmed that some lncRNAs were also upregulated in GI cancers [Table 2]. They are generally able to control the expression levels of certain proteins by sponging specific miRNAs, thus promoting chemoresistance of GI cancer cells to 5-FU^[78-101]^.

**Table 2:** LncRNAs that promote 5-FU resistance in GI cancers

**lncRNAs**	**Cancer type**	**Expression**	**Target or pathway**	**Ref.**
UCA1	CRC	up	miR-204-5p/Bcl-2, CREB1	[78]
UCA1	CRC	up	miR-23b-3p/ZNF281	[79]
NORAD	CRC	up	miR-495-3p/HIF-1α	[80]
TUG1	CRC	up	miR-197-3p/TYMS	[81]
HOTAIR	CRC	up	miR-218/ NF-kB	[82]
H19	CRC	up	miR-194-5p/SIRT1	[83]
PCAT6	CRC	up	miR-204/HMGA2	[84]
LINC02418	CRC	up	miR-372-3p/EPHA2	[85]
GAS6-AS1	CRC	up	PCBP1	[86]
HNF1A-AS1	GC	up	miR-30b-5p/EIF5A2	[87]
SNHG16	GC	up	miR-506-3p/PTBP1	[88]
PVT1	GC	up	Bcl-2, caspase3	[89]
HIT000218960	GC	up	HMGA2	[90]
OVAAL	GC	up	PCB	[91]
SUMO1P3	GC	up	CNBP	[92]
LINC02323	GC	up	miR-139-3p	[93]
XIST	HCC	up	miR-219/ SMC4	[94]
HULC	HCC	up	miR-6825-5p, miR-6845-5p, miR-6886-3p/ USP22	[95]
LINC00680	HCC	up	miR-568/ AKT3	[96]
Lnc-PKD2-2-3	CCA	up	miR-328/GPAM	[97]
FALEC	CCA	up	miR-20a-5p/ SHOC2	[98]
TUG1	PC	up	miR-376b-3p/ DPYD	[99]
HOTAIR	EC	up	MTHFR	[101]

5-FU: 5-fluorouracil; GI: gastrointestinal; lncRNAs: long non-coding RNAs; CRC: colorectal cancer; PC: pancreatic cancer; HCC: hepatocellular carcinoma; GC: gastric cancer; EC: esophageal cancer; CCA: cholangiocarcinoma; Bcl-2: B-cell lymphoma-2; CREB1: CAMP responsive element binding protein 1; ZNF281: zinc finger protein 281; HIF-1α: hypoxia-inducible factor-1a; TYMS: thymidylate synthetase; NF-kB: nuclear factor-kappa B; SIRT1: sirtuin 1; HMGA2: high mobility group AT-Hook 2; EPHA2: EPH receptor A2; PCBP1: poly (rc) binding protein 1; EIF5A2: eukaryotic translation initiation factor 5A2; PTBP1: polypyrimidine tract binding protein 1; PCB: pyruvate carboxylase; CNBP: cellular nucleic acid binding protein; SMC4: structural maintenance of chromosomes 4; USP22: ubiquitin-specific peptidase 22; GPAM: glycerol-3-phosphate acyltransferase, mitochondrial; SHOC2: SHOC2 leucine rich repeat scaffold protein; DPYD: dihydropyrimidine dehydrogenase; MTHFR: methylenetetrahydrofolate reductase.

#### CRC

The lncRNA UCA1 was able to sponge endogenous miR-204-5p and hinder its function, thereby improving the expression of Bcl-2 and CAMP responsive element binding protein 1 (CREB1), which ultimately suppressed apoptosis of CRC cells and increased 5-FU resistance^[78]^. Moreover, the lncRNA UCA1 could also sponge miR-23b-3p, with the consequent augmentation of zinc finger protein 281 (ZNF281) expression, which gives rise to the promotion of autophagy and the suppression of apoptosis in CRC cells^[79]^. It was observed that lncRNA H19 could function as ceRNA to sponge miR-194-5p and then enhance the expression of the downstream target sirtuin 1 (SIRT1), which induces the onset of autophagy in CRC^[80]^. Zhang *et al*. found that CRC cells exposed to hypoxia generally exhibited a stronger ability to develop resistance to 5-FU. Mechanistically, they confirmed that lncRNA NORAD could act as a ceRNA of miR-495-3p and finally upregulate the expression of HIF-1α^[81]^. It has been confirmed that the overexpression of TYMS was closely associated with 5-FU resistance. Wang *et al*. demonstrated that the lncRNA TUG1 negatively regulated miR-197-3p, which led to increased expression of TYMS and ultimately made CRC cells resistant to 5-FU^[82]^. Li *et al*. discovered that HOTAIR could suppress the expression of miR-218 in CRC by recruiting enhancer of zest homolog 2 (EZH2), which caused activation of nuclear factor-kappa B (NF-kB) signaling and ultimately repression of 5-FU-induced cytotoxicity in CRC cells^[83]^. Wu *et al*. found that overexpression of the lncRNA PCAT6 restrained the expression of miR-204, which improves high mobility group AT-Hook 2 (HMGA2)/PI3K signaling activity and ultimately enhances the chemoresistance^[84]^. Yao *et al*. disclosed that the upregulated lncRNA LINC02418 augmented 5-FU chemoresistance by sponging miR-372-3p and potentiating EPH receptor A2 (EPHA2) expression within CRC cells^[85]^. GAS6-AS1 could directly bind to PCBP1[poly (rc) binding protein 1] and enhance the expression of MCM3, thus promoting the development of 5-FU resistance. The combination of *GAS6-AS1* gene knockout and 5-FU chemotherapy drugs can produce better curative effects^[86]^.

#### GC

In GC cells, the elevated expression of the lncRNA HNF1A-AS1 has been demonstrated to enhance 5-FU resistance in GC cells by facilitating EMT via modulation of the miR-30b-5p/eukaryotic translation initiation factor 5A2 (EIF5A2) axis^[87]^. GC cells overexpressing polypyrimidine tract binding protein 1 (PTBP1) were found to have an increased rate of glycolysis and 5-FU resistance, but miR-506-3p was able to curb this process by directly targeting PTBP1. However, overexpression of lncRNA SNHG16 would downregulate miR-506-3p, thereby increasing PTBP1 expression and promoting 5-FU resistance^[88]^. Du *et al*. demonstrated that the lncRNA PVT1 could impede 5-FU-induced apoptosis in GC cells by upgrading Bcl-2 expression, increasing the Bcl-2/Bax ratio, and decreasing the expression of downstream cleaved caspase-3^[89]^. Another lncRNA, HIT000218960, exerted an inhibitory effect on 5-FU-induced apoptosis in GC cells by upregulating the expression level of HMGA2 and triggering the activation of the AKT/mTOR/P70S6 kinase (P70S6K) signaling pathway^[90]^. Tan *et al*. revealed that the lncRNA OVAAL could stabilize pyruvate carboxylase (PCB) and accelerate oxaloacetate-aspartate production, thereby promoting the synthesis of pyrimidine nucleotides. Finally, increased levels of dTMP protected GC from 5-FU-induced cell death^[91]^. The lncRNA SUMO1P3, which could interact with the cellular nucleic acid binding protein (CNBP), exhibited an upregulated status in GC. This upregulation enabled it to exert a positive regulatory influence on the downstream oncogenes c-Myc and cyclin D1 of CNBP, consequently facilitating 5-FU resistance^[92]^. It has been recently reported that LINC02323 was upregulated in GC. Silencing LINC02323 could upregulate its expression by negatively regulating miR-139-3p, thereby inducing apoptosis of GC cells after 5-FU treatment and increasing the sensitivity to 5-FU^[93]^.

#### HCC

It was shown that the lncRNA XIST could prevent miR-219 from reducing the expression of structural maintenance of chromosomes 4 (SMC4) and thus promoting autophagy and 5-FU resistance in HCC cells through the activation of the AMPK/mTOR signaling pathway^[94]^. Another lncRNA, namely HULC, can upregulate the expression of ubiquitin-specific peptidase 22 (USP22) through the suppression of miR-6825-5p, miR-6845-5p, and miR-6886-3p. This molecular cascade subsequently obstructs the degradation of SIRT, which finally leads to autophagy and 5-FU resistance in HCC cells^[95]^. Shu *et al*. disclosed that lncRNA LINC00680 could sponge miR-568 to accelerate the expression of AKT3 and activate its downstream signaling molecules such as mTOR, Eukaryotic translation initiation factor 4E (eIF4E) binding protein 1 (EIF4EBP1), and p70S6K, which induces 5-FU resistance of HCC cells^[96]^.

#### Other cancer types

Glycerol-3-phosphate acyltransferase, mitochondrial (GPAM) is the binding target of miR-328 in CCA cells. However, the overexpressed lnc-PKD2-2-3 could directly bind to miR-328, thereby positively regulating GPAM expression, and ultimately promoting 5-FU resistance in CCA cells^[97]^. LncRNA FALEC was ascertained to exhibit overexpression in CCA cells and augment the resistance of CCA cells to 5 - FU. This may be because FALEC could sponge miR-20a-5p and downregulate its expression, which in turn leads to an elevation in the expression of SHOC2 leucine-rich repeat scaffold protein (SHOC2)^[98]^.

Enhanced concentrations of DPYD commonly result in augmented degradation of 5-FU, thereby eliciting resistance of cancer cells to 5-FU. Tasaki *et al*. found that the lncRNA TUG1 significantly increased DPYD mRNA and DPD protein expression by suppressing miR-376b-3p in pancreatic ductal adenocarcinoma (PDAC) cells and thus contributed to 5-FU chemoresistance^[99]^. Methylenetetrahydrofolate reductase (MTHFR), which directly manages DNA methylation, has been shown to increase the 5-FU sensitivity of CRC cells^[100]^. However, Zhang *et al*. found that the lncRNA HOTAIR reduced MTHFR expression by promoting DNA methylation of the MTHFR proponent, thereby favoring the resistance of EC cells to 5-FU^[101]^.

### CircRNAs that promote 5-FU resistance in GI cancers

CircRNAs can also promote 5-FU resistance in GI cancers [Table 3]^[102-106]^. Circ_0000338, existing within extracellular vesicles, was capable of augmenting 5-FU resistance in CRC. It achieved this by diminishing apoptosis via binding to miR-217 and miR-485-3p and exerting a negative regulatory effect on their expression^[102]^. Circ_0032833 induced positive regulation of Musashi RNA-binding protein 1 (MSI1) by sponging miR-125-5p and thus reducing the 5-FU sensitivity of CRC cells^[103]^.

**Table 3:** CircRNAs that promote 5-FU resistance in GI cancers

**circRNAs**	**Cancer type**	**Expression**	**Target or pathway**	**Ref.**
circ_0000338	CRC	up	miR-217, miR-485-3p	[102]
circ_0032833	CRC	up	miR-125-5p/MSI1	[103]
circNRIP1	GC	up	miR-138-5p/HIF-1α	[104]
circCPM	GC	up	miR-21-3p/PRKAA2	[105]
circ_0003998	HCC	up	miR-513a-5p/ARK5	[106]

5-FU: 5-fluorouracil; GI: gastrointestinal; circRNAs: circular RNAs; CRC: colorectal cancer; HCC: hepatocellular carcinoma; GC: gastric cancer; MSI1: Musashi RNA Binding Protein 1; HIF-1α: hypoxia-inducible factor-1a; PRKAA2: protein kinase AMP-activated catalytic subunit alpha 2; ARK5: AMPK-related protein kinase 5.

In GC cells, HIF-1α is one of the most important metabolic regulators of glycolysis. Xu *et al*. found that circNRIP1 raised HIF-1α-dependent glucose metabolism by sponging miR-138-5p, thereby enhancing hypoxia-induced resistance to 5-FU in GC cells^[104]^. The protein kinase AMP-activated catalytic subunit alpha 2 (PRKAA2) can initiate the autophagy process. Fang *et al*. confirmed that circCPM can induce 5-FU chemoresistance of GC by targeting miR-21-3p, which enhances autophagy by increasing PRKAA2 translation^[105]^. Recently, Xue *et al*. found that circ_0003998 could sponge miR-513a-5p and boost the level of AMPK-related protein kinase 5 (ARK5), thereby elevating 5-FU resistance in HCC cells^[106]^.

## NCRNAS INHIBIT 5-FU RESISTANCE IN GI CANCERS

### MiRNAs that inhibit 5-FU resistance in GI cancers

In contrast to the miRNAs described above, some miRNAs are able to increase the 5-FU sensitivity of GI cancer cells. However, they were usually downregulated in cancer cells [Table 4]^[107-129]^.

**Table 4:** MiRNAs that inhibit 5-FU resistance in GI cancers

**miRNAs**	**Cancer type**	**Expression**	**Target or pathway**	**Ref.**
miR-96	CRC	down	XIAP, UBE2N	[107]
miR-143	CRC	down	ERK5, NF-kB	[108]
miR-206	CRC	down	Bcl-2	[109]
miR-129	CRC	down	Bcl-2	[110]
miR-218	CRC	down	BIRC5	[111]
miR-22	CRC	down	BTG1	[112]
miR-375-3p	CRC	down	TYMS	[113]
miR-203	CRC	down	TYMS	[114]
miR-494	CRC	down	DPYD	[115]
miR-122	CRC	down	PKM2	[116]
miR-200b-3p	CRC	down	ZEB1, E2F3	[117]
miR-145	CRC	down	RAD18	[118]
miR-761	CRC	down	FOXM1	[119]
miR-302b	HCC	down	Mcl-1, DPYD	[120]
miR-125b	HCC	down	HK II	[121]
miR-27a	HCC	down	FZD7	[122]
miR-503	HCC	down	eIF4E	[123]
miR-204	GC	down	TGFBR2	[124]
miR-139-5p	GC	down	HOXA13	[125]
miR-138-5p	PC	down	VIM	[126]
miR-137	PC	down	PTN	[127]
miR-106b	CCA	down	ZBTB7A	[128]
miR-338-5p	EC	down	Id-1	[129]

5-FU: 5-fluorouracil; GI: gastrointestinal; miRNAs: microRNAs; CRC: colorectal cancer; PC: pancreatic cancer; HCC: hepatocellular carcinoma; GC: gastric cancer; EC: esophageal cancer; CCA: cholangiocarcinoma; XIAP: X-linked inhibitors of apoptosis; UBE2N: ubiquitin-conjugating enzyme E2N; ERK5: extracellular signal-regulated kinase 5; NF-kB: nuclear factor-kappa B; Bcl-2: B-cell lymphoma-2; BIRC5: baculoviral IAP repeat containing 5; BTG1: B-cell translocation gene 1; TYMS: thymidylate synthetase; DPYD: dihydropyrimidine dehydrogenase; PKM2: pyruvate kinase M2; ZEB1: zinc finger E-box binding homeobox 1; E2F3: E2F transcription factor 3; RAD18: RAD18 E3 ubiquitin protein ligase; FOXM1: Forkhead Box M1; Mcl-1: MCL1 apoptosis regulator; HK II: Hexokinase II; FZD7: Frizzled homolog protein 7; eIF4E: eukaryotic translation initiation factor 4E; TGFBR2: transforming growth factor beta receptor 2; HOXA13: Homeobox A13; VIM: vimentin; PTN: pleiotrophin; ZBTB7A: zinc finger and BTB domain containing 7A; MTPN: myotrophin; Id-1: inhibitor of DNA binding 1.

#### CRC

X-linked inhibitors of apoptosis (XIAP) and the ubiquitin-conjugating enzyme E2N (UBE2N) are known for their role as negative regulators of apoptosis. Kim *et al*. found that increased levels of miR-96 in CRC cells led to increased apoptosis after exposure to 5-FU by decreasing their expression^[107]^. Another miRNA, miR-143, could downregulate the expression of extracellular signal-regulated kinase 5 (ERK5) and NF-kB in CRC cells, leading to an enhancement of pro-apoptotic effects and ultimately increasing the sensitivity to 5-FU^[108]^. MiR-206 and miR-129 were found that they could suppress Bcl-2 protein synthesis specifically, thus promoting CRC cell apoptosis reduced by 5-FU^[109,110]^. Furthermore, another recent study has demonstrated that miR-218 was capable of enhancing the apoptosis reduction induced by 5-FU in CRC through the suppression of baculoviral IAP repeat containing 5 (BIRC5)^[111]^. B-cell translocation gene 1 (BTG1) is capable of inducing cellular autophagy. However, Zhang *et al*. discovered that miR-22 could obstruct autophagy and enhance apoptosis in CRC cells treated with 5-FU^[112]^.

As for 5-FU metabolism, Xu *et al*. disclosed that miR-375-3p could augment the sensitivity of CRC cells to 5-FU by inducing apoptosis and cell cycle arrest via the direct suppression of TYMS^[113]^. Similarly, Li *et al*. found that overexpression of miR-203 could enhance 5-FU chemosensitivity by downregulating TYMS^[114]^. The tumor-inhibiting factor miR-494 is capable of directly binding to DPYD and exerting a negative regulatory effect on its expression, thereby enhancing the apoptosis of CRC cells induced by 5-FU^[115]^. In terms of glucose metabolism, miR-122 could hinder the expression of pyruvate kinase M2 (PKM2) and its associated glycolysis process in CRC cells, thereby decreasing 5-FU resistance^[116]^.

It has been confirmed that hypoxic conditions could elevate the secretion of exosomes by tumor cells, which modulate the TME and contribute to the emergence of drug resistance^[117]^. Zinc finger E-box binding homeobox 1 (ZEB1) and E2F transcription factor 3 (E2F3) have been identified as direct targets of exosomal miR-200b-3p. The dearth of miR-200b-3p in exosomes originating from hypoxic cancer-associated fibroblasts (CAFs) leads to an enhancement resistance to 5-FU treatment in the CRC cells^[59]^.

In cancer cells, RAD18 E3 ubiquitin protein ligase (RAD18) plays a key role in DNA damage repair. However, miR-145 could negatively regulate RAD18, thereby enhancing chemosensitivity to 5-FU by promoting DNA damage in CRC cells^[118]^. Cao *et al*. revealed that miR-761 could directly bind to forkhead box M1 (FOXM1) and negatively regulate its expression to enhance the 5-FU chemosensitivity of CRC^[119]^.

#### HCC

MCL1 apoptosis regulator (Mcl-1) plays a crucial role in impeding the apoptosis of cancer cells. Interestingly, miR-302b could suppress the expression of Mcl-1 and increase the apoptosis of HCC cells in response to 5-FU treatment. Furthermore, miR-302b could also suppress the expression of DPYD^[120]^. Hexokinase II (HK II) plays an important role in glycolysis. MiR-125 could directly target HK II to downregulate glucose metabolism and sensitize HCC cells to 5-FU^[121]^. Frizzled homolog protein 7 (FZD7) can upgrade the expression of P-glycoprotein (P-gp). However, miR-27a could downregulate FZD7 expression and repress the β-catenin pathway, thereby reversing the P-gp-mediated 5-FU chemoresistance^[122]^. eIF4E is closely associated with tumor growth, invasion, and metastasis. Interestingly, miR-503 could negatively regulate the expression of eIF4E and increase the sensitivity of HCC cells to 5-FU^[123]^.

#### Other cancer types

It was determined that miR-204 can heighten the sensitivity of GC cells to 5-FU chemotherapy. Mechanistically, miR-204 could impede the TGF-β-mediated EMT by targeting the transforming growth factor beta receptor 2 (TGFBR2)^[124]^. Homeobox A13 (HOXA13), which is a member of the HOX family, gives rise to the resistance of GC patients to 5-FU treatment. However, HOXA13 could be negatively regulated by miR-139-5p, thereby alleviating the drug resistance to 5-FU^[125]^. Yu *et al*. found that the overexpression of miR-138-5p increased the chemosensitivity of PC cells to 5-FU. Yu *et al*. discovered that the overexpression of miR-138-5p enhanced the chemosensitivity of PC cells to 5-FU. The possible reason for this is the direct targeting of vimentin (VIM) by miR-138-5p, resulting in its downregulation, which ultimately curbed the EMT process^[126]^. It was found that miR-137 increased the 5-FU sensitivity of PC cells, possibly because of the significant downregulation of pleiotrophin (PTN) through miR-137 overexpression^[127]^. Zinc finger and BTB domain containing 7A (ZBTB7A) has been characterized as an oncogene across numerous cancer types. However, as Jiao *et al*. uncovered, ZBTB7A could serve as a direct target of miR-106b in CCA cells. The induction of miR-106b augments cellular sensitivity to 5-FU, whereas the restoration of forced ZBTB7A expression counteracts this effect^[128]^. In ESCC cells, the inhibitor of the DNA binding 1 gene (*Id-1*) was often overexpressed and linked to a poor prognosis. MiR-338-5p, being downregulated in ESCC, was capable of sensitizing ESCC cells to 5-FU treatment. It could achieve this by directly targeting *Id-1* and subsequently downregulating its expression^[129]^.

### LncRNAs that inhibit 5-FU resistance in GI cancers

Some lncRNAs could also enhance the 5-FU sensitivity of GI cancer cells [Table 5]^[130-135]^. The overexpression of lncRNA HAND2-AS1 could decrease miR-20a expression, leading to the upregulation of PDCD4 expression and enhancing 5-FU-induced apoptosis in CRC cells^[130]^. Another lncRNA, ENST00000547547, was able to directly bind to miR-31 and repress its expression. This action promotes 5-FU-induced apoptosis and diminishes 5-FU chemoresistance in CRC^[131]^.

**Table 5:** LncRNAs that inhibit 5-FU resistance in GI cancers

**lncRNAs**	**Cancer type**	**Expression**	**Target or pathway**	**Ref.**
HAND2-AS1	CRC	down	miR-20a/PDCD4	[130]
ENST00000547547	CRC	down	miR-31	[131]
KRAL	HCC	down	miR-141/ KEAP1	[132]
SNHG16	HCC	down	has-miR-93	[133]
GAS5	PC	down	miR-181c-5p/MST1	[134]
LINC00261	EC	down	DPYD	[135]

5-FU: 5-fluorouracil; GI: gastrointestinal; lncRNAs: long non-coding RNAs; CRC: colorectal cancer; PC: pancreatic cancer; HCC: hepatocellular carcinoma; EC: esophageal cancer; PDCD4: programmed cell death factor 4; KEAP1: Kelch-like ECH-associated protein 1; MST1: macrophage stimulating 1; DPYD: dihydropyrimidine dehydrogenase.

A study carried out by Wu *et al*. initially showed that lncRNA KRAL can compete with KEAP1 mRNA to bind miR-141. This binding promotes KEAP1 expression and blocks the Nrf2/ARE pathway, ultimately improving 5-FU sensitivity in HCC cells^[132]^. In contrast to the increased expression of SNHG16 in GC, Xu *et al*. found that SNHG16 level was notably reduced in HCC. Moreover, they found that SNHG16 could significantly constrain 5-FU chemoresistance by competitively binding to hsa-miR-93^[133]^.

As for other types of cancers, Gao *et al*. revealed that miR-181c-5p could reduce the level of MST1 to suppress the Hippo signaling in PC cells. However, the upregulation of lncRNA GAS5 could negatively regulate miR-181c-5p, resulting in a rise in the expression level of MST1. This activation of the Hippo signaling pathway improves 5-FU resistance^[134]^. Lin *et al*. disclosed that lncRNA LINC00261 could enhance the chemosensitivity of EC cells to 5-FU. Mechanistically, LINC00261 could bind to the DPYD promoter region and enhance the regulation of DPYD methylation, thereby suppressing the expression of DPYD^[135]^.

## THE IMPLICATION OF 5-FU RESISTANCE-RELATED NCRNAS IN GI CANCERS

5-FU is rarely used alone at present, but is most commonly used as a basic component of chemotherapy regimens such as FOLFOX. Among patients suffering from metastatic CRC and demonstrating resistance to 5-FU-centered chemotherapy regimens, the expression of miR-130b, miR-106a, and miR-484 in plasma was higher. Given that the plasma samples were collected prior to treatment initiation, it can be inferred that these miRNAs hold the potential to function as reliable predictive biomarkers for gauging the resistance to 5-FU-based chemotherapy regimens^[136]^. Another report showed that GC cancer patients with increased expressions of miR-191 and miR-425 in serum had an increased risk of being insensitive to the FOLFOX regimen, and both miRNAs could serve as diagnostic markers for chemotherapy resistance to this regimen in patients with advanced GC cancer^[137]^. Another miRNA, miR-320e, can act as a new prognostic biomarker, and its upregulated expression usually predicts an unfavorable outcome for patients with stage III CRC who are undergoing 5-FU adjuvant chemotherapy^[138]^. In addition, lncRNA XIST also has the potential to become a potential biomarker for 5-FU resistance in GI cancers, and its upregulation usually indicates a poor response to 5-FU treatment^[139]^. The above studies have demonstrated that ncRNAs have great potential to serve as markers for measuring the resistance of GI cancer patients to 5-FU or 5-FU-based chemotherapy regimens. However, it is worth noting that most of the current studies have focused on individual plasma samples or other body fluid samples and the detection of a single ncRNA therein, which limits the persuasiveness of the detection accuracy. Considering combined assays based on tissues and sera, as well as combined analysis of multiple ncRNAs, may be a better solution.

Currently, there are two main approaches commonly used in tumor treatments targeting ncRNAs. One is to inhibit specifically overexpressed ncRNA molecules in tumors, which can be achieved by using antisense oligonucleotides (ASO), siRNA, miRNA sponges, CRISPR/Cas9-based genome editing, small molecule inhibitors targeting ncRNAs, *etc*. The other is to enhance the tumor-suppressive effects by overexpressing tumor-suppressive ncRNAs^[21]^. It can be inferred that by inhibiting those ncRNAs whose expressions are upregulated and promote 5-FU resistance in GI cancers or increasing those ncRNAs whose expressions are downregulated and can enhance the sensitivity of tumor cells to 5-FU, it may be possible to effectively overcome the 5-FU resistance in GI cancers. Fortunately, some studies have already attempted to achieve this by focusing on ncRNAs. Yu *et al*. found that treating drug-resistant GC cancer SGC7901 cells with Rosmarinic acid (RA) could downregulate the expressions of miR-6785-5p and miR-642a-3p, thereby enhancing the chemical sensitivity to 5-FU^[140]^. In another report, Wang *et al*. utilized GCD nanocarriers to co-deliver 5-FU and siRNA fragments (Anti-miRNA-10b) that could specifically inhibit miR-10b, effectively targeting CRC tumors with overexpressed EGFR, inhibiting the growth of CRC cancer cells and overcoming the problem of 5-FU resistance^[141]^. More interestingly, a modified 5-FU-miR-15a was constructed by guiding the replacement of uracil (U) residues on the miR-15a strand with 5-FU. Compared with 5-FU, its inhibitory effect on PDAC cells was enhanced^[142]^. This miRNA-based method of incorporating 5-FU into the nucleic acid strand for chemical modification may be used to treat other types of GI cancers and overcome their 5-FU resistance problems in the future.

## CONCLUSION

5-FU plays a significant role in cancer chemotherapy as a crucial anti-tumor medication. 5-FU can achieve anti-tumor effects by interfering with the synthesis of DNA or RNA in tumor cells, making it a broad-spectrum anti-tumor agent, particularly effective in digestive tract cancers. However, the development of 5-FU chemoresistance in GI cancers significantly impacts the curative effect of cancer treatment and the patients' prognostic situation. Therefore, exploring the molecular regulatory mechanism of drug resistance and discovering potential targets for intervention is one of the urgent challenges to be addressed today.

An increasing number of studies have demonstrated that ncRNAs are of vital importance in the development and regulation of 5-FU drug resistance in GI cancers. At present, targeting of ncRNAs can be achieved by curbing ncRNA expression, promoting ncRNA degradation, and altering the interaction between ncRNAs and their targets. This approach offers a new perspective for addressing the issue of 5-FU drug resistance. Therefore, it is necessary to summarize and elaborate on the ncRNAs involved in regulating 5-FU resistance in GI cancers.

**Figure 1:**
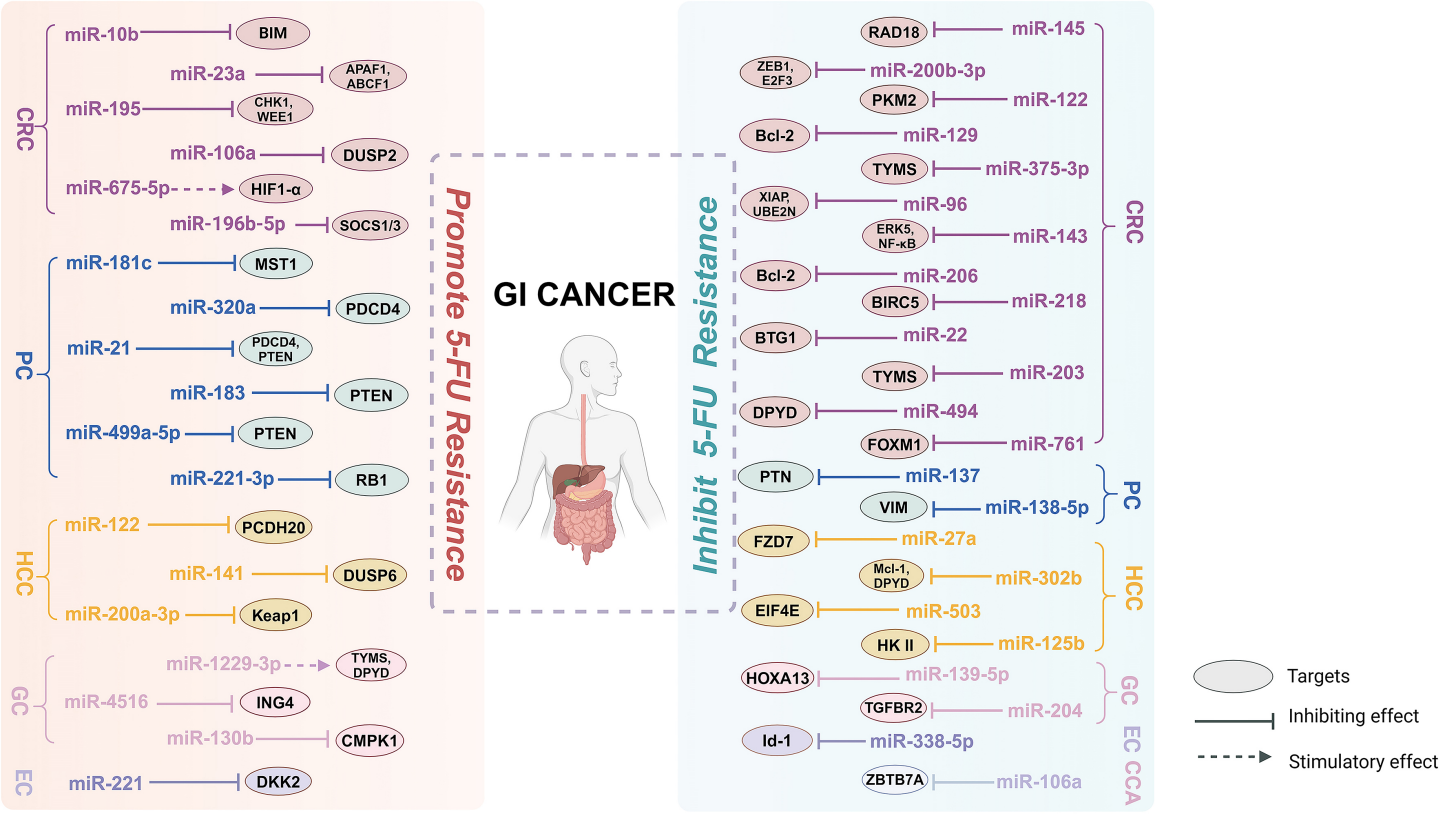
MiRNAs that regulate 5-FU resistance in GI cancers. 5-FU: 5-fluorouracil; GI: gastrointestinal; miRNAs: microRNAs; CRC: colorectal cancer; PC: pancreatic cancer; HCC: hepatocellular carcinoma; GC: gastric cancer; EC: esophageal cancer; CCA: cholangiocarcinoma; BIM: Bcl-2-interacting mediator of cell death; CHK1: checkpoint kinase 1; WEE1: WEE1 G2 checkpoint kinase; HIF-1α: hypoxia-inducible factor-1α; MST1: macrophage stimulating 1; PDCD4: programmed cell death factor 4; PTEN: phosphatase and tensin homolog; PCDH20: protocadherin 20; KEAP1: Kelch-like ECH-associated protein 1; ING4: Inhibitor of growth family member 4; DKK2: dickkopf WNT signaling pathway inhibitor 2; DUSP2: dual specificity phosphatase 2; SOCS1: suppressor of cytokine signaling 1; SOCS3: suppressor of cytokine signaling 3; RB1: RB corepressor 1; DUSP6: dual-specificity phosphatase 6; CMPK1: cytidine/uridine monophosphate kinase 1; TYMS: thymidylate synthase; DPYD: dihydropyrimidine dehydrogenase; ZEB1: zinc finger E-box binding homeobox 1; E2F3: E2F transcription factor 3; Bcl-2: B-cell lymphoma-2; XIAP: X-linked inhibitors of apoptosis; BTG1: B-cell translocation gene 1; PTN: pleiotrophin; FZD7: Frizzled homolog protein 7; EIF4E: eukaryotic translation initiation factor 4E; HOXA13: Homeobox A13; Id-1: inhibitor of DNA binding 1; RAD18: RAD18 E3 ubiquitin protein ligase; PKM2: pyruvate kinase M2; FOXMI: Forkhead Box M1; VIM: vimentin; Mcl-1: MCL1 apoptosis regulator; HK II: Hexokinase II; TGFBR2: transforming growth factor beta receptor 2; ZBTB7A: zinc finger and BTB domain containing 7A.

**Figure 2:**
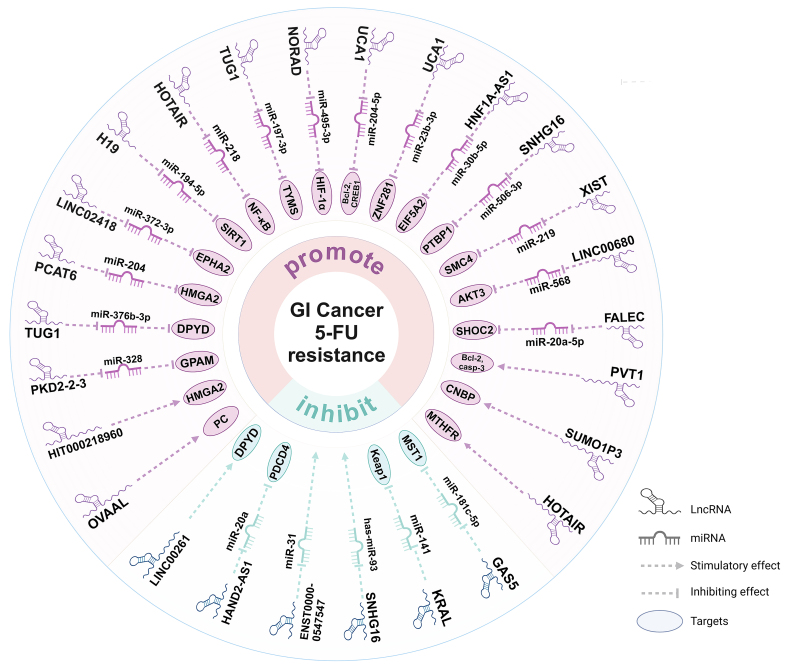
LncRNAs that regulate 5-FU resistance in GI cancers. GI: Gastrointestinal; 5-FU: 5-fluorouracil; lncRNAs: long non-coding RNAs; miRNAs: microRNAs; CNBP: cellular nucleic acid binding protein; MTHFR: methylenetetrahydrofolate reductase; HMGA2: high mobility group AT-Hook 2; GPAM: glycerol-3-phosphate acyltransferase, mitochondrial; DPYD: dihydropyrimidine dehydrogenase; EPHA2: EPH receptor A2; SIRT1: sirtuin 1; NF-kB: nuclear factor-kappa B; TYMS: thymidylate synthase; HIF-1α: hypoxia-inducible factor-1α; Bcl-2: B-cell lymphoma-2; CREB1: CAMP responsive element binding protein 1; ZNF281: zinc finger protein 281; EIF5A2: eukaryotic translation initiation factor 5A2; PTBP1: polypyrimidine tract binding protein 1; SMC4: structural maintenance of chromosomes 4; SHOC2: SHOC2 leucine rich repeat scaffold protein; MTHFR: methylenetetrahydrofolate reductase; MST1: macrophage stimulating 1; KEAP1: Kelch-like ECH-associated protein 1; PDCD4: programmed cell death factor 4; PCB: pyruvate carboxylase.

**Figure 3:**
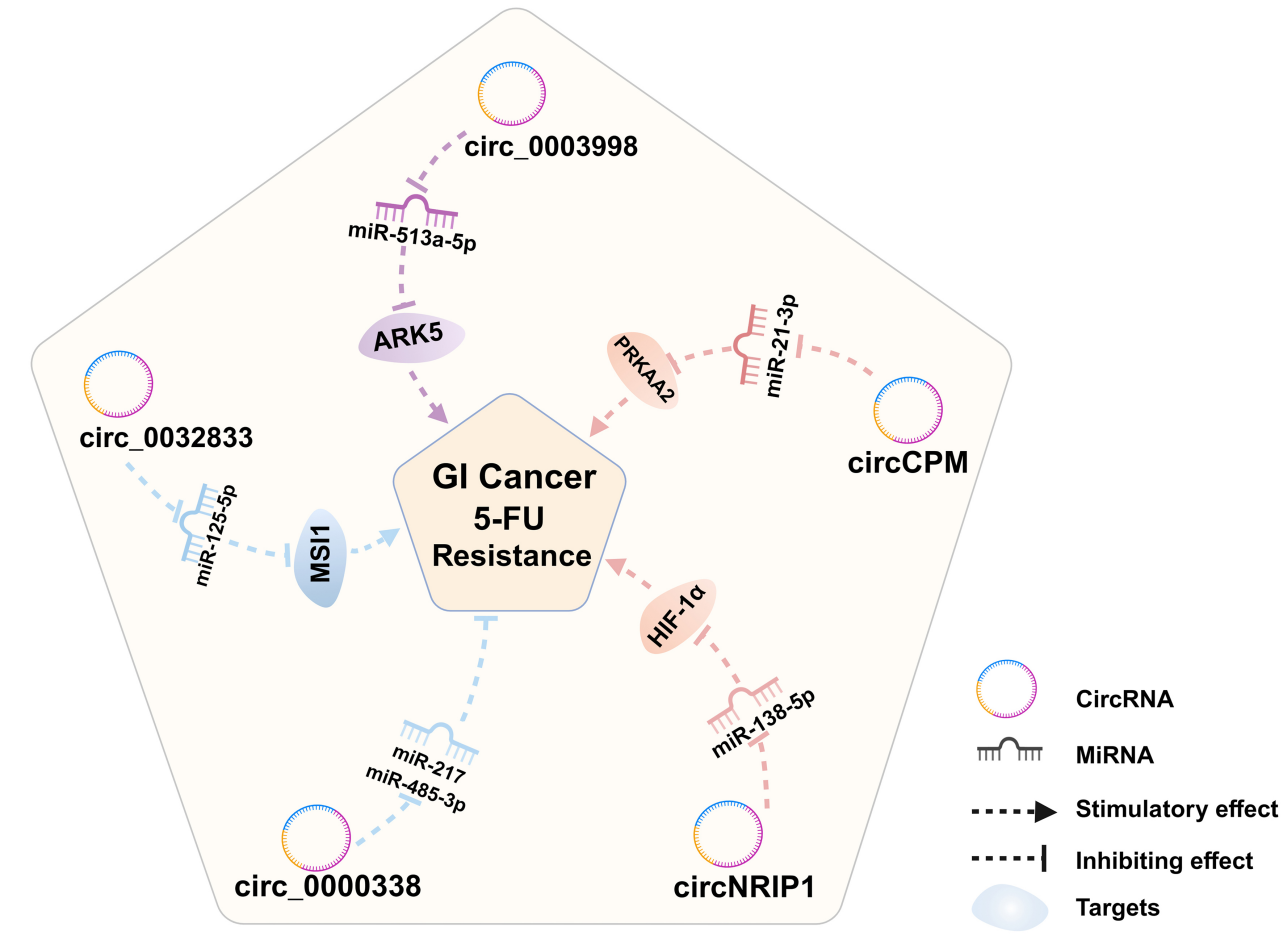
CircRNAs that regulate 5-FU resistance in GI cancers. GI: Gastrointestinal; 5-FU: 5-fluorouracil; circRNAs: circular RNAs; ARK5: AMPK-related protein kinase 5; MSI1: Musashi RNA Binding Protein 1; HIF-1α: hypoxia-inducible factor-1α; PRKAA2: protein kinase AMP-activated catalytic subunit alpha 2.

In this paper, the regulatory targets and mechanisms of three types of ncRNAs, miRNAs [Figure 1], lncRNAs [Figure 2], and circRNAs [Figure 3] in 5-FU drug resistance in GI cancers have been introduced. The effect of ncRNAs on the emergence of 5-FU resistance is readily apparent, as they can modulate various pathways. For example, tumor cells mediate changes in TME through ncRNAs to protect themselves from the destruction of chemotherapy drugs. In addition, ncRNAs also regulate 5-FU resistance by affecting EMT, apoptosis, TS levels, 5-FU metabolism, autophagy, glucose metabolism, and drug efflux. Notably, some ncRNAs raise the emergence of 5-FU resistance; conversely, some ncRNAs also increase the sensitivity of GI cancer cells to 5-FU chemotherapy. In addition, the regulatory effects of the same ncRNA on different types of cancers vary. For instance, the lncRNA SNHG16 mentioned in the paper can enhance the progression of 5-FU resistance in GC cells, whereas its function is contradictory in HCC cells. The differences in the regulatory role of SNHG16 on 5-FU resistance may depend on the location and microenvironment of specific cancer types as well as the different miRNA targets it sponges. It can be seen that the regulation of 5-FU drug resistance by ncRNAs has dual roles, and it is complex and diverse.

Apparently, numerous ncRNAs play important roles in regulating the 5-FU resistance of GI cancers. It is worth noting that some ncRNAs also have significant applications in actual clinical treatments, especially in terms of serving as biomarkers in predicting, treating, and assessing prognosis for 5-FU resistance in GI cancers. In addition, researchers are also attempting to start with non-coding RNAs (ncRNAs) in order to overcome 5-FU resistance. Despite the significant role of ncRNAs in overcoming resistance to 5-FU chemotherapy, treatment strategies focusing on a single ncRNA may be ineffective because of the intricate nature of cancer signaling pathways and targets. Consequently, integrating the modulation of ncRNAs with conventional radiotherapy, targeted therapy, and immunotherapy might represent the most favorable strategy to augment efficacy and surmount 5-FU resistance in the future.

## DECLARATIONS

### Copyright

© The Author(s) 2025.
